# Supplementary material for: Comprehensive Annotation and Functional Exploration of MicroRNAs in Lettuce
Source: Front Plant Sci. 2021 Dec 24;12:781836. doi: 10.3389/fpls.2021.781836 (PMC8739914; doi:10.3389/fpls.2021.781836)
Supplement: Supplementary file 2 [file Data_Sheet_8.ZIP › GSM2230750.plot/Lsa-miR167c_Lsat_1_v5_gn_7_36161.1_520_TPlot.pdf]

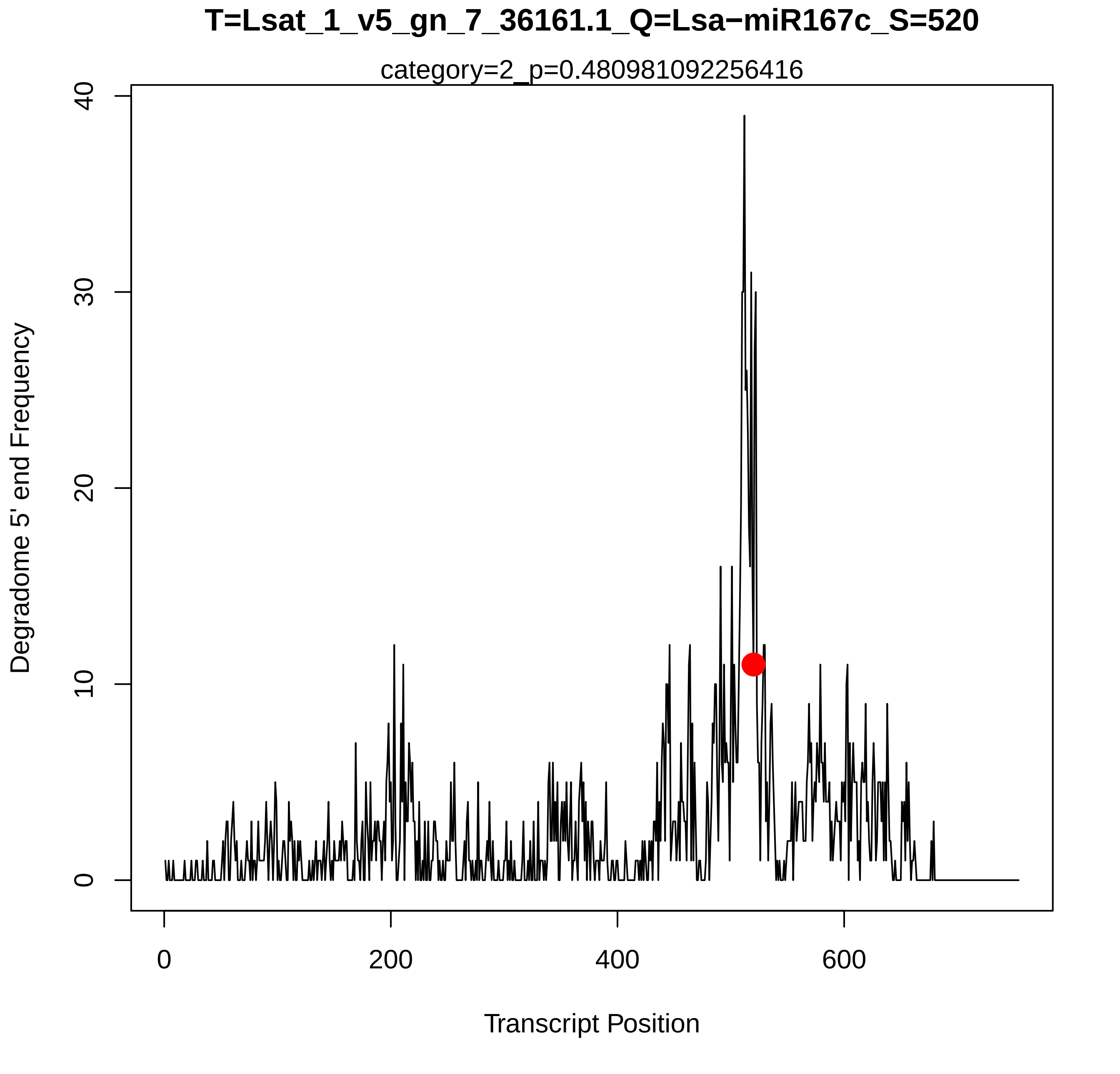
T=Lsat_1_v5_gn_7_36161.1_Q=Lsa−miR167c_S=520
category=2_p=0.480981092256416
Degradome 5' end Frequency
0
10
20
30
40
0
200
400
600
Transcript Position
